# Effects of Diet on Larval Survival, Growth, and Development of the Sea Cucumber *Holothuria leucospilota*

**DOI:** 10.1155/2022/8947997

**Published:** 2022-10-08

**Authors:** Zonghe Yu, Hong Wu, Youkai Tu, Zesen Hong, Jiewen Luo

**Affiliations:** College of Marine Sciences, South China Agricultural University, Guangzhou, China

## Abstract

Because most tropical sea cucumbers have been overexploited around the world, the sea cucumber *Holothuria leucospilota* has become increasingly commercially important in recent years. Restocking and aquaculture of *H. leucospilota* using hatchery-produced seeds could both enhance declining wild populations and provide sufficient beche-de-mer product to satisfy increasing market demand. Identifying an appropriate diet is important for successful hatchery culture of the *H. leucospilota*. In this study, we trialed different ratios of microalgae *Chaetoceros muelleri* (2.00–2.50 × 10^6^ cells/mL) and yeast (*Saccharomyces cerevisiae*, ~2.00 × 10^6^ cells/mL) in diets for *H. leucospilota* larvae (6 d after fertilization, referred to as “day 0”) at proportions 4 : 0, 3 : 1, 2 : 2, 1 : 3, and 0 : 4 by volume, in 5 treatments (A, B, C, D, and E, respectively). Larval survival rates in these treatments decreased over time, with the survival highest in treatment B (59.24 ± 2.49%) on day 15 (double that of the lowest rate in treatment E (28.47 ± 4.23%)). For any sampling event, larval body length in treatment A was always the lowest after day 3, and that for treatment B was always the highest, except on day 15. The maximum percentage of doliolaria larvae occurred in treatment B (23.33%) on day 15, followed by treatments C, D, and E (20.00%, 10.00%, and 6.67%, respectively). No doliolaria larvae occurred in treatment A, and pentactula larvae occurred only in treatment B (3.33%). On day 15 in all treatments, late auricularia larvae had hyaline spheres, but these were not prominent in treatment A. Densities of juveniles attaching to settlement plates varied with treatments, and the values were very low for the larvae only fed microalgae (A, 2.39 ± 1.95 ind per plate) or yeast (E, 2.13 ± 1.05 ind per plate)—only ~5% of the maximum number settling in treatment B (45.56 ± 7.24 ind per plate). Increased larval growth, survival and development, and juvenile attachment indicates that diets combining microalgae and yeast are more nutritionally balanced than single diets for hatchery of *H. leucospilota.* A combined diet of *C. muelleri* and *S. cerevisiae* at a 3 : 1 ratio is optimum for the larvae. Based on our results, we propose a larval rearing protocol to facilitate mass production of *H. leucospilota*.

## 1. Introduction

Throughout Asia, sea cucumbers are considered both a delicacy and a medicinal food. Their high market demand, coupled with their sedentary nature, late age at maturity, slow growth, and low rates of recruitment render most sea cucumber species vulnerable to overexploitation, with their depletion possibly leading to indirect, deleterious effects on marine ecosystems [1–6].

The widely distributed edible sea cucumber *H. leucospilota* occurs in shallow tropical and subtropical Indo-Pacific Ocean waters, where it forms an important part of traditional subsistence fisheries for some Pacific Island nations [1, 3, 5, 7, 8]. This species is also a major source of biologically active substances, such as polysaccharides and saponins for biotechnology and medicine, which further increases its market value [5, 8].

This species plays an important role in nearshore marine ecosystem because it moves large amounts of sand and selectively feeds on organically rich components in the sediment. It is involved in exchange of nutrients and dissolved oxygen between the water column and sediment surface and alters the sediment biota and carbonate dynamics of its habitat. Because this species can endure a wide temperature range, and its coculture in aquaculture systems mitigates the effects of organic pollution (improving environmental quality), it is an ideal species to use in integrated multitrophic aquaculture (IMTA) systems [3, 7–10].

*Holothuria leucospilota* has been severely overexploited and affected by environmental degradation [3, 4, 7] Throughout Asia it is fished in China, Malaysia, Thailand, Indonesia, the Philippines, and Vietnam, and where it is fished, there are few regulations pertaining to its harvesting [3, 7]. Because it is accessible and population recruitment is slow, it is particularly vulnerable to overfishing, and once its stocks have decreased dramatically, they can take years to recover [2, 6]. Both its overexploitation and the lack of harvest regulations in Malaysia could jeopardize *H. leucospilota* locally [4]. We have reported a population of this species at the mouth of a bay in China to have decreased by 90% over 4 years as a consequence of fishing pressure [7].

Restocking, sea ranching, and aquaculture of sea cucumbers using hatchery-produced seeds could both enhance declining wild sea cucumber populations and provide sufficient beche-de-mer product to satisfy increasing market demand [2, 5, 11, 12]. Several sea cucumber species (e.g., *Apostichopus japonicus* and *H. scabra*) have been artificially bred and reared at a commercial scale [13–17]. However, culture of *H. leucospilota* is presently only experimental [5, 15].

Identifying suitable diets is the most important step for sea cucumber hatchery. Diets should be high-quality and relatively inexpensive and not be difficult or sophisticated to culture [17–20]. Traditional diets for rearing sea cucumber larvae have mainly used live microalgae. Although single-species microalgal diets can be effective for rearing larvae of some holothurian species [12, 19, 21–24], diets comprising multiple microalgae species are considered to be more nutritionally balanced and have been recommended for sea cucumber hatchery to improve larval survival and growth [13, 19, 25]. Unfortunately, microalgal culture is expensive, accounting for about 30% of the total operational hatchery costs, so maintaining multiple microalgae species would be even more expensive [26]. More cost-effective diets that yield high production and quality of sea cucumber larvae must be identified.

*Chaetoceros* sp. as a diet has yielded good results for growth and survival of holothurian larvae [22, 25]. The microalga *Chaetoceros muelleri* can tolerate a high temperature, salinity, photoperiod, and light intensity range and is characterized by fast growth and easy maintenance [24, 26–28]. Because this species is highly nutritious, it is often used as a food in aquaculture, and it is widely used in sea cucumber hatcheries for species such as *A. japonicus*, *H. scabra*, *Australostichopus mollis*, and *Stichopus horrens* [13, 20, 23, 24, 29–31]. The alga *C. muelleri* could represent an integral component of the diet for rearing *H. leucospilota* larvae [5, 15].

Characteristics of substitute diets include that they are highly nutritious, inexpensive, easy to use, more stable than live microalgal cultures, and free of pests and/or diseases that could cause mass mortality of cultured species [18, 31–33]. While ground shrimp pellets and fine *Sargassum* spp. paste are effective for growing late auricularia larvae of *H. scabra* [12], commercially available microalgae concentrates could also be used to replace live microalgae for this species [18, 34, 35] and for *A. japonicas* [31]. Baker's (dry) yeast *Saccharomyces cerevisiae* and red yeast *Rhodotorula benthica*, both rich in protein and vitamins, are also acceptable phytoplankton substitutes for *A. japonicus* larvae [20, 31–33] .

Optimal diets for sea cucumber larvae are species-specific [36]. Although a mixture of microalgae (*C. muelleri*) and yeast (*S. cerevisiae*) was reportedly efficient for rearing *H. leucospilota* larvae [5], neither the amount nor proportion of either was specified. We provide *C. muelleri* or *S. cerevisiae*, or different mixtures of the two planktonic larval *H. leucospilota*, to assess the varied effects of diet on water quality and the growth, survival, and development of larvae and juvenile attachment to identify an optimum diet regimen for mass production of this species.

## 2. Materials and Methods

### 2.1. Broodstock Maintenance and Larvae Production

Broodstock of *H. leucospilota* was collected on SCUBA from ~5-m depth in Daya Bay (22°33′N, 114°33′E), Guangdong, southern China, on 14 August 2020. Animals were transported to the Marine Biology Research Station Laboratory at Daya Bay and acclimated in 400-L tanks containing aerated, sand-filtered seawater (passed through 5*-μ*m filter prior to usage) at 29°C for 2 days to empty their guts. Feces were siphoned from tanks twice daily, and seawater was changed daily. Healthy broodstock >200 g was washed individually to remove external sediment and organisms before being placed into reproduction tanks.

Animals were dry stimulated [13] to induce spawning. Eggs from 5 females and sperm from 2 males were mixed; fertilized eggs were siphoned onto a 100-*μ*m sieve placed into a bucket, washed several times with clean seawater to remove excess sperm, and then transferred to 1000-L larval rearing tanks. Five air stones were placed on the bottom of each tank to provide uniform aeration and to distribute the eggs; larval density was 1.6 larvae/mL. Two days after fertilization, when the auricularia larval stage was reached, larvae were fed a mixture of microalgae *C. muelleri* (15,000 cells/mL) and yeast (*S. cerevisiae*) powder (0.5 g/m^3^) and 50% of the seawater replaced daily [5]. Six days after fertilization, when larvae developed into the mid-auricularia stage, they were collected for diet experimentation. The larval survival rate during this period was about 95%.

### 2.2. Experimental Diets

#### 2.2.1. Chaetoceros muelleri

The *C. muelleri* stock was obtained from the Institute of Oceanology, Chinese Academy of Sciences. The *C. muelleri* used in the experiment was grown at 27–30°C under continuous white LED lights using standard f/2 medium with silicon [37]; underwater aeration was provided to keep algae in suspension. Cultures during the exponential growth phase were harvested to feed sea cucumber larvae. Cells were counted under an optical microscope (Eclipse E200, Nikon, Japan) using a hemocytometer, with densities used in experiments ranging 2.00–2.50 × 10^6^ cells/mL.

#### 2.2.2. Saccharomyces cerevisiae

Baker's (dry) yeast *S. cerevisiae* (Harbin Mauri Yeast Co., Ltd., Harbin, China) was used as substitute diet for larvae. Preweighed yeast powder was placed into a 100*-μ*m mesh bag and soaked; the yeast paste was then homogenized by pressing it into a certain volume of seawater. The concentration of yeast suspension used in experiments was 0.10 g/L (~2.00 × 10^6^ cells/mL).

### 2.3. Diet Experiments

Diet experiments commenced on 22 August 2020, 6 days postfertilization, hereinafter referred to as “day 0.” Larvae of *H. leucospilota* were collected and randomly allocated into one of 15 circular tanks (400 L) with clean seawater. Initial larval body length was 598.49 ± 31.04 *μ*m (mean ± SD, *n* = 15), and the initial stocking density in each tank was 0.65 larvae/mL. Five microalgal/yeast diet regimens were tested at ratios (by volume) of 4 : 0, 3 : 1, 2 : 2, 1 : 3, and 0 : 4, in treatments A, B, C, D, and E, respectively. Three replicate tanks were used for each treatment. For the first 9 days, larvae were fed in the morning and afternoon with a specific diet at a rate of 5 L/m^3^; 30% of the seawater was replaced in each tank each afternoon, prior to the second feeding. The daily ration was doubled to 10 L/m^3^ per feeding event after day 9, when late auricularia larvae were first observed, and 50% of the seawater was replaced daily. Penicillin was added at a rate of 2 g/m^3^ every 3 days after water renewal. Experiments were performed under natural photoperiod, with light intensity <1000 lux at the water surface.

Experiments were run for 15 days. Samples of 20 mL (*n* = 5) from each tank were collected to determine larval survival every 3 days (days 3, 6, 9, 12, and 15) before water was replaced each afternoon. Growth was also determined every 3 days (excepting day 6) by measuring the mean body length of 10 randomly selected larvae from each replicate tank. On day 15, larvae at different developmental stages were counted, and their stages were expressed as a percentage of the total number of live individuals to indicate metamorphosis rate. Individuals in different treatments continued to be fed using aforementioned diets until no planktonic larvae were observed in the water column, usually about 3 days afterwards.

Larvae were observed and measured using an optical microscope (Eclipse E200, Nikon, Japan). Water parameters were measured for each sampling event: water temperature (°C), salinity, and pH were monitored using an In-Situ smarTROLL Multiparameter Handheld instrument (In-Situ Inc., Fort Collins, CO, USA), and nitrite (NO_2_-N; mg/L) concentration was analyzed following Grasshoff et al. [38].

### 2.4. Juvenile Attachment

When nonfeeding doliolaria larvae were observed, 3 sets of settlement plates were placed into each tank upon which they could settle and metamorphose. Each set of plates comprised 20 pieces of wavy PVC (31 cm × 39 cm) coated with benthic diatoms *Nitzschia* sp. and *Navicula* sp. (plates coated with these two diatoms provide an optimal substratum upon which holothurian larvae can settle [39]). After attachment, juveniles were fed twice daily with a combined feed for juvenile sea cucumber *A. japonicus* (comprising *Sargassum* spp., scallop apron, soybean meal, yeast, *Spirulina* sp., vitamins, and trace elements; with crude protein, crude lipid, crude fiber, and ash contents of 14%, 6%, 15%, and 45%, respectively). The amount of food was regulated by juvenile density and size; 30% of the seawater was replaced daily.

Juvenile *H. leucospilota* density was estimated on 1 November 2020, 77 days after fertilization. Juveniles attached to plates were counted, and their density calculated as numbers of juveniles on five randomly selected plates from each tank.

### 2.5. Statistical Analysis

Statistical analysis was performed using IBM SPSS Statistics for Windows, version 19.0 (IBM Corp., Armonk, N.Y., USA). Data from different treatments for each sampling period were analyzed by one-way ANOVA, followed by comparisons of means using the Student–Newman–Keuls (SNK) test. Data (as percentages) were arcsine transformed to obtain normality prior to analysis but are presented as nontransformed data [8]. Statistical significance was set at *p* < 0.05.

## 3. Results

### 3.1. Water Parameters

**Figure 1:**
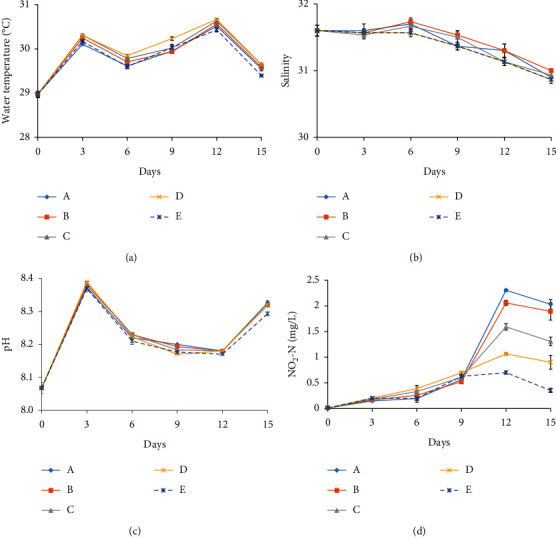
Water parameters in treatments (a–d) (a) temperature, (b) salinity, (c) pH, and (d) NO2-N. Data are presented as mean ± SD (*n* = 3). Diets comprising *Chaetoceros muelleri* and *Saccharomyces cerevisiae* in each treatment are mixed in proportions of 4 : 0 A, 3 : 1 B, 2 : 2 C, 1 : 3 D, and 0 : 4 E by volume.

Water temperature ranged 28.98–30.67°C during experimentation (Figure 1(a)). Salinity was relatively stable (30.87–31.73) (Figure 1(b)), but in all treatments decreased slightly after day 6 because of rainfall. pH ranged 8.07–8.39, with values in different treatments comparatively high on days 3 and 15 (Figure 1(c)). Water temperature, salinity, and pH in different treatments were very similar for any sampling event.

Initial NO_2_-N concentrations were very low (0.01 ± 0.00 mg/L) on day 0 and increased steadily with time to day 9 (Figure 1(d)), when values in treatments were 0.56 ± 0.08 (A), 0.52 ± 0.03 (B), 0.60 ± 0.03 (C), 0.70 ± 0.02 (D), and 0.63 ± 0.04 mg/L (E). Values for treatments A–C, with high proportions of *C. muelleri*, increased remarkably after day 9 (when diet was doubled), and NO_2_-N concentrations in treatments A–E reached 2.31 ± 0.02 (A), 2.06 ± 0.05 (B), 1.59 ± 0.06 (C), 1.06 ± 0.02 (D), and 0.70 ± 0.03 mg/L (E) on day 12. NO_2_-N concentrations in all treatments decreased slightly on the last sampling event, although values in descending order remained A > B > C > D > E.

### 3.2. Survival

Survival rates of larvae fed different diets are presented in Table 1. Survival decreased in all treatments over time, with the survival in treatments D and E significantly lower than for other treatments on day 3. On day 9 treatment E had the lowest value (64.67 ± 2.89%) and treatment C had the highest (80.08 ± 4.06%). There was no significant difference among any treatment on days 6 and 12 of sampling (*p* = 0.100 and *p* = 0.239, respectively). On day 15, the survival rate varied significantly among treatments (*p* = 0.001), and the value of larvae in treatment B fed *C. muelleri* and *S. cerevisiae* at a 3 : 1 proportion was the highest (59.24 ± 2.49%) and double that of the lowest rate for treatment E (28.47 ± 4.23%) for which larvae were fed only yeast; there was no significant difference among values in other treatments for this sampling event.

### 3.3. Growth

**Figure 2:**
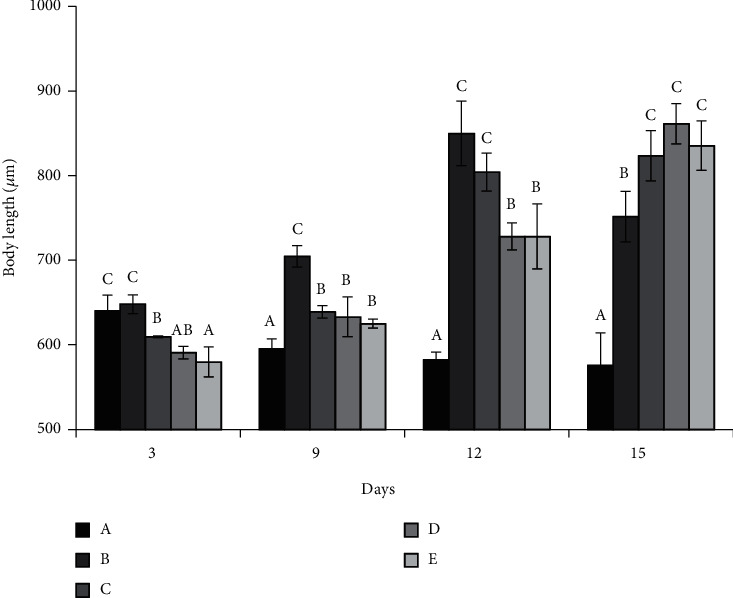
Variation in body length of *Holothuria leucospilota* larvae in different diet treatments. Data are presented as means ± SD (*n* = 3). For a given sampling event, different letters above bars indicate significant differences among treatments.

Larval body length in different treatments is shown in Figure 2. Lengths in treatment A increased from day 0 to 3 before decreasing to an average 575.90 ± 38.11 *μ*m on day 15. Lengths in treatment B increased over time to peak (849.78 ± 38.00 *μ*m) on day 12, then decreased to 751.48 ± 29.83 *μ*m on day 15. Lengths in treatment C increased with time and peaked (823.22 ± 29.73 *μ*m) on day 15. Lengths in treatments D and E decreased from day 0 to 3, then increased to peak on day 15 (861.26 ± 23.73 and 835.30 ± 29.32 *μ*m, respectively). There were always significant differences among the values of the five treatments for each sampling event (all *p* < 0.05). After day 3, for any given sampling event, larvae in treatment A always had the lowest body length, while individuals in treatment B were always the largest, excepting on day 15. Larval body length in treatments C–E was significantly higher than those in treatments A and B on day 15; there was no significant difference among values in these three treatments.

### 3.4. Development

**Figure 3:**
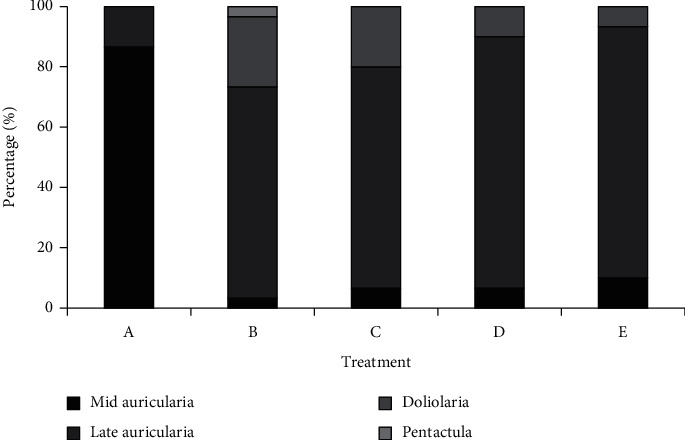
Proportional composition of *Holothuria leucospilota* larval stages in different diet treatments on day 15. *n* = 30.

Development of larvae in different treatments on day 15 (21 days after fertilization) is shown in Figure 3. The percentage of mid-auricularia stage larvae (apparent arrested development) in treatments A, B, C, D, and E is 86.67%, 3.33%, 6.67%, 6.67%, and 10.00%, respectively, while 13.33%, 70.00%, 73.33%, 83.33%, and 83.33% of larvae had reached a late auricularia stage, respectively. The maximum percentage of doliolaria larvae occurred in treatment B (23.33%), followed by C (20.00%), D (10.00%), and E (6.67%); no doliolaria larvae were observed in treatment A. Pentactula larvae occurred only in treatment B (3.33%), with the percentage of metamorphosed larvae (doliolaria and pentactula) in this treatment (26.66%) the highest among treatments. Body lengths of doliolaria and pentactula larvae were approximately half those of late auricularia stage larvae, and the high percentage of metamorphosed larvae in treatment B rendered mean body length lower than for treatments C–E.

**Figure 4:**
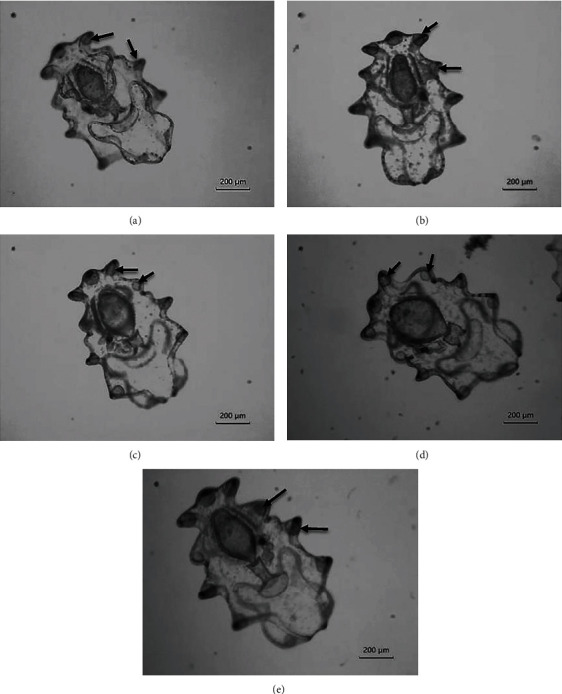
Late auricularia larvae of *Holothuria leucospilota* on day 15 in different diet treatments: (a) A, (b) B, (c) C, (d) D, and (e) E. Arrows indicate hyaline spheres.

All late auricularia larvae observed in treatments on day 15 had hyaline spheres (Figure 4), although spheres in larvae in treatment A (Figure 4(a)) were not as prominent as in other treatments.

### 3.5. Juvenile Attachment

**Figure 5:**
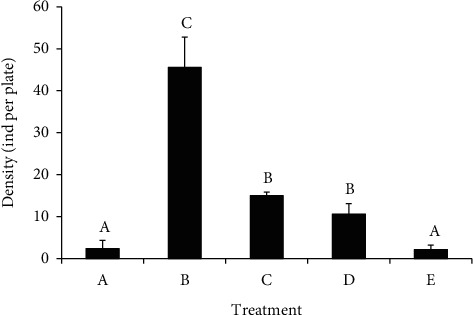
Density of *Holothuria leucospilota* juveniles on settlement plates in different diet treatments. Data are presented as means ± SD (*n* = 3). Different letters above bars indicate significant differences among treatments.

Densities of juveniles attached to settlement plates differed significantly among treatments (*p* < 0.001) (Figure 5). Low densities occurred in the treatments in which larvae were only fed microalgae (A, 2.39 ± 1.95 ind per plate) or yeast (E, 2.13 ± 1.05 ind per plate); their densities in these two treatments were only ~5% of the maximum value reported for treatment B (45.56 ± 7.24 ind per plate). Densities in treatments C and D were 15.00 ± 0.87 and 10.61 ± 2.47 ind per plate, respectively—only ~33% or 23% the values for treatment B. There was no significant difference between values for treatments A and E or C and D.

## 4. Discussion

Water quality plays an important role in sea cucumber hatchery because larvae are highly sensitive to environmental change [22, 40]. Water temperature, salinity, and pH in our culture system were relatively consistent and generally within normal ranges for rearing larvae of tropical holothurian species [5, 11, 13, 22, 24]. However, NO_2_-N concentrations decreased with the proportion of yeast in diets. The microalgal culture medium and nitrogenous waste (including uneaten food, fecal matter, metabolic excretions, and other compounds) in the culture environment can be converted into NO_2_-N by bacteria. This deteriorates water quality and increases NO_2_-N concentrations, which are toxic to many aquatic organisms. Reduced survival, growth, and development of cultured animals, especially their larvae, is reported at high NO_2_-N concentrations [31, 41–45]. For crustaceans, NO_2_-N concentrations significantly affect larval freshwater prawn *Macrobrachium rosenbergii* survival, with death occurring at concentrations as low as 3.3 mg/L to 8 days; the sublethal concentration was 1.4 mg/L over 7 days, but growth was significantly affected [41] . Safe NO_2_-N concentrations for rearing juvenile Pacific white shrimp *Litopenaeus vannamei* are estimated as 6.1, 15.2, and 25.7 mg/L at salinities of 15, 25, and 35, respectively [44], and lower (1.36 mg/L) for postlarval tiger prawn *Penaeus monodon* [42]. The toxic effects of NO_2_-N on echinoderms vary among species, with safe limits (no influence on growth) for the sea urchin *Paracentrotus lividus* being 1–2 mg/L [46], and a negative effect occurring on the gonad index of *Strongylocentrotus droebachiensis* at 0.5 mg/L [47]. The specific growth rate of *A. japonicus* decreased with increased NO_2_-N concentration, with a “safe level” being 10.00, 19.54, and 27.70 mg/L for individuals of wet weight of 2.91, 48.96, and 102.54 g, respectively [45]. To our knowledge, no information is available on “safe levels” of NO_2_-N concentrations for holothurian larvae.

Water quality can be controlled by water renewal, with partial water changes usually adopted for larger larval tanks to reduce temperature-induced stress [5, 13, 17]. However, rearing water is often renewed completely in small-scale experiments [15, 19, 22]. We partially exchange 30% to 50% of the water on a daily basis in our tanks, with the proportion of water exchanged being much lower than earlier studies involving *H. leucospilota* larvae [5, 15]. Our larval survival rates on day 15 in treatments B–D are higher than that of Huang et al. [5] (<30% when larvae reached the doliolaria stage). We cannot infer that delayed growth and development of *H. leucospilota* larvae in treatment A (with the highest NO_2_-N concentration) was because of poor water quality, because individuals in treatment B (which also had high NO_2_-N concentrations) performed better than other treatments, and the survival rate of larvae in treatment E (with the lowest NO_2_-N concentrations) was the lowest among treatments. Accordingly, we deem the water replacement protocol in our study to have been suitable for rearing *H. leucospilota* larvae. NO_2_-N concentrations are easily measured and do indicate accumulation of nitrogenous waste in the breeding system, but to determine the influence of NO_2_-N on *H. leucospilota* larvae, and the optimum water renewal strategy, requires further study.

Rearing density affects the success of holothurian larval culture, and suitable densities are species-specific. For example, 0.5–1 larvae/mL is considered to be ideal for raising larval *H. scabra* [17, 39, 48], and 0.1–0.5 larvae/mL is suggested for larval *A. japonicas* [31, 48, 49]. Our stocking density (0.65 larvae/mL) appears to be suitable for rearing *H. leucospilota* because larval survival, growth, and development in treatments B–D were similar to or better than those at 0.2–0.5 larvae/mL [5], or 0.15 larvae/mL [15]. Further investigation on the effects of rearing density is required to more precisely determine optimum stocking densities.

Because diet quantity and composition are important to regulate growth, survival, and development of holothurian larvae, an optimal diet is important for their culture [6, 23, 25]. Microalgal diet concentrations administered to larvae of tropical holothurians are usually maintained at ~20,000–40,000 cells/mL [6, 12, 13, 15, 17]. Lower concentrations can be applied if the diet is augmented with a supplementary feed [5]. Quantities of *C. muelleri* and/or *S. cerevisi*ae fed to *H. leucospilota* larvae (20,000–50,000 cells/mL) in this study are generally within optimum ranges, although larval performance vary depending on the diet regimen.

Some larval holothurian species prefer a single-species diet. For example, *Chaetoceros calcitrans* is effective for rearing larval *Bohadschia marmorata* [21], *H. spinifera* [22], and *Parastichopus californicus* [19], and dry yeast *S. cerevisi*ae or sea yeast *R. benthica* can be used as stable food for *A. japonicus* larvae [20, 31, 33]. However, multispecies diets are usually used to rear holothurian larvae [5, 13, 17, 20, 29, 30, 50]. Combined diets are generally more nutritionally balanced and efficient than single diets. For example, the metamorphosis rate of *H. spinifera* larvae fed a combined *Isochrysis galbana + C. calcitrans* diet was higher (100%) than those fed solely with *I. galbana* (40%) or *C. calcitrans* (85%) [22]; *H. scabra* larvae fed a mixed diet grew and developed better than those fed single species diets [25, 36]; and *P. californicus* larva fed a mix of microalgae containing *C. calcitrans* had higher survival, growth, and metamorphosis rates [19]. Although *C. muelleri* can provide all nutritional requirements for larval *A. japonicas* [31], *A. mollis* [23], and *H. scabra* [24] growth and development, this is not so for *H. leucospilota*. Our results indicate that mixed diets are better for rearing larval *H. leucospilota*, because larvae fed a combined microalgal/yeast diet had improved survival, growth, and development than those fed solely *C. muelleri* or *S. cerevisiae*.

Polyunsaturated fatty acids (PUFA), especially docosahexaenoic acid (DHA) and eicosapentaenoic acid (EPA), are essential components of the diet of marine invertebrates. PUFA levels in the diet correlate significantly with larval performance of *H. scabra* [18, 34]. According to results of the previous studies, the microalgae always contain higher proportions of PUFA than the baker's yeast [26, 51, 52]. The PUFA, DHA, and EPA levels of *C. muelleri* were 23.93, 1.25, and 12.95% of total fatty acid, respectively [52], while those of *S. cerevisi*ae were 5.74, 0.05, and 0.25%, respectively [51]; therefore, in the current study, the diet in treatment A had the highest PUFA, DHA, and EPA levels among all treatments. However, larvae in this treatment performed worse than those fed a combined diet, so PUFA is not a good indicator of the nutritional value of a diet for *H. leucospilota* larvae, consistent with findings for larval *P. californicus* [19]. The nutritional requirements of *H. leucospilota* larvae remain poorly known. Other nutrient components such as proteins, carbohydrates, and vitamins may play a more important role.

The planktonic larvae of most holothurian species require fewer than 15–18 days to reach the nonfeeding doliolaria stage, e.g., 8–9 d for *H. polii* [6], 9–10 d for *Stichopus* sp. [11], 10–12 d for *H. spinifera* [50], and 13–14 d for *H. scabra*, *Pseudocolochirus violaceus*, and *Colochirus quadrangularis* [12]. Because the planktonic larval stage of *H. leucospilota* is quite long [5, 15], it is desirable to shorten this because prolonged larval development is less economically viable in culture, and larvae are more susceptible to infections from pests and diseases. We report a yeast diet to increase development of larval *H. leucospilota*, with larvae partly fed *S. cerevisiae* in treatments B–D, or entirely in treatment E reaching doliolaria and even pentactula stages within 21 d of fertilization. This is similar to Huang et al. [5], wherein yeast was included in the diet, and faster than when larvae were solely fed *C. muelleri* (treatment A herein), or a mixture of microalgae (*C. muelleri*, *C. calcitrans*, and *Tetraselmis* sp.), in which larvae reached the doliolaria stage in 22–27 d postfertilization [15].

Development of hyaline spheres is a prominent characteristic of late auricularia stage Aspidochirotida holothurian larvae prior to their metamorphosis, which depends heavily on larval diet [36]. Hyaline spheres likely function as nutritive reserves for holothurian larvae and play a role in buoyancy; the presence and size of these spheres indicates larval competence [19, 23, 35, 36, 53]. Poorly developed hyaline spheres in late auricularia larvae in treatment A indicate inadequate nutritive reserves, which negatively affect larval metamorphosis and juvenile attachment. Although hyaline spheres were prominent in late auricularia stage larvae in treatment E, which were only fed yeast, larval settlement in this treatment was relatively low. The potentially unbalanced nature of this single diet, along with low larval survival, might contribute to poor settlement.

Standard procedures and guidelines have been developed to improve the growth, survival, and development of larvae and juveniles of economically important holothurian species (e.g., *A. japonicus*, *H. scabra*, and *Isostichopus fuscus*) [13, 16, 20]. Despite this, comparable information for *H. leucospilota* is unavailable. A simple, practical, and reliable method to mass produce *H. leucospilota* larvae is necessary. A fixed-dose procedure is more beginner-friendly and useful for sea cucumber hatchery. An effective larval rearing protocol for mass production of *H. leucospilota* might entail an initial stocking density of ~0.6 larvae/mL, with planktonic larvae fed a combined diet of the microalga *C. muelleri* and yeast *S. cerevisiae* (with *C. muelleri* in an exponential growth phase at ≥2.00 × 10^6^ cells/mL, and powdered *S. cerevisiae* homogenized in seawater to a concentration of ~2.00 × 10^6^ cells/mL) mixed at a 3 : 1 ratio (by volume), fed to larvae twice daily (morning and afternoon). This diet could be first provided at 5 L/m^3^ per feeding event, accompanied by a 30% change in the culture water each day prior to the second feeding. When late auricularia stage larvae occur, the diet could be doubled, and 50% of the seawater could be replaced daily. When nonfeeding doliolaria stages occur, a substratum coated with benthic diatoms could be introduced into tanks for larval settlement and metamorphosis. Seawater for the larval rearing system could be sand-filtered, and passed through 5 *μ*m filter prior to usage; culture water could be continuously aerated, with temperature, salinity, and pH maintained within normal ranges for rearing tropical holothurian larvae. An antibiotic (e.g., penicillin) could be added at a rate of 2 g/m^3^ every 3 days after water renewal. While this protocol is preliminary, further investigation is required to determine optimal water parameters, rearing density, nutritional requirements, feeding protocols, and even settlement cues for *H. leucospilota* larvae.

## 5. Conclusion

In conclusion, diets combining microalgae and yeast are more nutritionally balanced than single diets for hatchery of *H. leucospilota*, and a combined diet of *C. muelleri* and *S. cerevisiae* at a 3 : 1 ratio is optimum for the larvae.

## Figures and Tables

**Table 1:** Temporal variation in survival rate of *Holothuria leucospilota* larvae in different diet treatments.

Treatments	Days				
	3	6	9	12	15
A	92.73 ± 3.08^b^	72.56 ± 0.83^a^	67.78 ± 9.20^a^	50.33 ± 1.96^a^	42.47 ± 1.85^b^
B	93.54 ± 2.21^b^	78.99 ± 7.40^a^	74.73 ± 6.23^ab^	61.14 ± 4.08^a^	59.24 ± 2.49^c^
C	93.81 ± 1.95^b^	86.97 ± 5.32^a^	80.08 ± 4.06^b^	58.69 ± 13.44^a^	49.46 ± 7.11^b^
D	83.13 ± 3.09^a^	75.03 ± 7.38^a^	68.09 ± 3.89^ab^	48.82 ± 2.43^a^	46.99 ± 8.21^b^
E	83.07 ± 5.03^a^	77.16 ± 7.29^a^	64.67 ± 2.89^a^	51.33 ± 8.42^a^	28.47 ± 4.23^a^

Data are presented as means ± SD (*n* = 3). For a given sampling event, different superscript letters above bars indicate significant differences among treatments.

## Data Availability

The data that support the findings of this study are available from the corresponding author upon reasonable request.
